# Android malware detection with MH-100K: An innovative dataset for advanced research

**DOI:** 10.1016/j.dib.2023.109750

**Published:** 2023-11-02

**Authors:** Hendrio Bragança, Vanderson Rocha, Lucas Barcellos, Eduardo Souto, Diego Kreutz, Eduardo Feitosa

**Affiliations:** a Institute of Computing, Federal University of Amazonas, Amazonas, Brazil; b Federal University of Pampa, Rio Grande do Sul, Brazil

**Keywords:** Android Malware, Android security, Malware detection, Machine learning

## Abstract

High-quality datasets are crucial for building realistic and high-performance supervised malware detection models. Currently, one of the major challenges of machine learning-based solutions is the scarcity of datasets that are both representative and of high quality. To foster future research and provide updated and public data for comprehensive evaluation and comparison of existing classifiers, we introduce the MH-100K dataset [1], an extensive collection of Android malware information comprising 101,975 samples. It encompasses a main CSV file with valuable metadata, including the SHA256 hash (APK's signature), file name, package name, Android's official compilation API, 166 permissions, 24,417 API calls, and 250 intents. Moreover, the MH-100K dataset features an extensive collection of files containing useful metadata of the VirusTotal1 analysis. This repository of information can serve future research by enabling the analysis of antivirus scan result patterns to discern the prevalence and behaviour of various malware families. Such analysis can help to extend existing malware taxonomies, the identification of novel variants, and the exploration of malware evolution over time.

Specifications Table

**Table:** 

Subject	Computer Science
Specific subject area	Data Mining and Machine Learning
Data format	Analysed, Filtered
Type of data	Figures, Tables, Malicious and Benign APK feature set
Data collection	We downloaded the APK files from Androzoo and the scanners' analysis from VirusTotal. Androzoo's data can be accessed through the following link: https://androzoo.uni.lu/access. The VirusTotal API overview is available at https://developers.virustotal.com/reference/overview.
Data source location	The data was collected from the extensive selection of Android applications accessible on Androzoo. The sampling duration extends over a span of 13 years, covering the timeframe from 2010 to 2022, resulting in a comprehensive global coverage.
Data accessibility	Data hosted in public repository. Repository name: Malware-Hunter dataset Direct URL to data: https://figshare.com/articles/dataset/MH-100K-Dataset/24328885

## 1 Value of the Data


 

• The dataset includes 101,975 Android malware samples, each represented by several features and class label, suitable for machine learning input.
• This dataset is valuable for machine learning, data mining, and computer security researchers, as well as practitioners. Enthusiasts in computer security can leverage the data to improve malware detection systems.
• This dataset can help in building more reliable classification models, offering easily extractable attributes that benefit both researchers and practitioners.
• Furthermore, the provided data can serve as a performance benchmark for advancing state-of-the-art machine learning methods in the field of malware classification.

## 2 Data Description

The MH-100 K dataset is an extensive repository containing 101,975 Android samples, with 9800 categorized as malicious applications using a threshold of at least 4 positive scanners from VirusTotal analysis. The primary component of this dataset is a central CSV file containing essential metadata, including the SHA256 hash (representing the APK's digital signature), file name, package name, Android's official compilation API, 166 permissions, 24,417 API calls, and 250 intents. Additionally, the MH-100K dataset encompasses a substantial collection of files that collectively store the results of the VirusTotal analysis [2].

This dataset offers valuable potential for future research, enabling the analysis of trends in antivirus scan results to gain deeper insights into the presence and behaviour of diverse malware families. Such analysis can facilitate the development of malware taxonomies, the identification of novel variants, and the study of malware evolution over time.

A description of the MH100K metadata data is provided in Table 1. The hexadecimal value of “SHA256” cryptographic hash is shown as a string. The “NAME” refers to the name of the application. The “PACKAGE” is a specific identification for each application. The “TARGET_API” denotes the API level against which the application has been tested, whereas the “MIN_API” identifies the lowest Android API level required for the program to function.

**Table 1:** List of metadata attributes and descriptions.

Metadata			
#	ATTRIBUTE	TYPE	DESCRIPTION
1	SHA256	String	SHA256 cryptographic hash
2	NAME	String	Package Name
3	PACKAGE	String	Unique Application ID
4	MIN_API	Numerical	Minimum API Level Required to Run
5	TARGET_API	Numerical	API Level That the Application Targets

Table 2 includes examples of app permissions, which refers to a set of rules that grant or deny activities such as access to specific features or data. Android uses this system to ensure that apps may only access data and conduct actions the user has expressly authorized. The complete list of permissions is available in the Malware Hunter repository. These permissions ensure that apps have access to device capabilities or data.

**Table 2:** Example of permissions included in MH-100K dataset.

Permissions		
#	ATTRIBUTE	DESCRIPTION
9	WRITE_SETTINGS	Allows an app to read or write the system settings
10	INTERNET	Allows applications to open network sockets.
2700	ACCESS_FINE_LOCATION	Allows an app to access precise location
2702	GET_ACCOUNTS	Allows access to the list of accounts in the Accounts Service
3251	CAMERA	Required to be able to access the camera device
3436	CHANGE_NETWORK_STATE	Allows applications to change network connectivity state
5100	SEND_SMS	Allows an application to send SMS messages
6381	WRITE_SECURE_SETTINGS	Allows an application to read or write the secure system settings
8194	INSTALL_PACKAGES	Allows an application to install packages
22,418	MANAGE_EXTERNAL_STORAGE	Allows an application a broad access to external storage in scoped storage
23,827	HIGH_SAMPLING_RATE_SENSORS	Allows an app to access sensor data with a sampling rate greater than 200 Hz

A second kind of feature is the intent actions, which can indicate application or system states such as completed boot or low battery. In Table 3 we provide a few examples of intent actions. Developers can register their apps to listen to these intents to ensure responsive and dynamic behaviour in line with the device's status or user actions. The complete list can be found in the repository.

**Table 3:** Example of Intents included in MH100K dataset.

Intents		
#	ATTRIBUTE	DESCRIPTION
11	AUDIO_BECOMING_NOISY	Broadcast intent, a hint for applications that audio is about to become 'noisy' due to a change in audio outputs
2708	BOOT_COMPLETED	It can be used to perform application-specific initialization
3264	WIFI_STATE_CHANGED	Broadcast intent action indicating that Wi-Fi has been enabled, disabled, enabling, disabling, or unknown
3442	BATTERY_LOW	Broadcast Action: Indicates low battery condition on the device
3959	MEDIA_MOUNTED	Broadcast Action: External media is present and mounted at its mount point
6295	PACKAGE_FULLY_REMOVED	Broadcast Action: An existing application package has been completely removed from the device
7601	DEVICE_ADMIN_ENABLED	This is the primary action that a device administrator must implement to be allowed to manage a device
10,069	ACTION_SCREEN_ON	Broadcast Action: Sent when the device wakes up and becomes interactive
13,673	LOGIN_ACCOUNTS_CHANGED	Sent when accounts are added, accounts are removed, or an account's credentials (saved password, etc.) are changed
18,147	DEVICE_OWNER_CHANGED	Broadcast action: sent when the device owner is set, changed or cleared
21,530	APPLICATION_RESTRICTIONS_CHANGED	Sent after application restrictions are changed

Table 4 shows a selection of a few API method calls from the Android platform, providing tools and functionality for developers to interface with various Android systems and device features. Android developers can utilize the methods and functions the Android system offers to create, enhance, or enable specific application features through API calls. Due to these calls, applications can engage with system components, access system resources, connect with other apps, or utilize specific functionality. In our dataset, we have 24,417 API calls.

**Table 4:** Example of API calls included in MH100K dataset.

API Calls		
#	ATTRIBUTE	DESCRIPTION
12	Landroid/content/Intent.touri()	Convert this Intent into a String holding a URI representation of it
29	Landroid/content/Context.getapplicationinfo()	Return the full application info for this context's package
123	Landroid/content/sharedpreferences.getboolean()	Retrieve a boolean value from the preference
112	Landroid/net/connectivitymanager.getactivenetworkinfo()	Returns details about the currently active default data network
659	Landroid/os/usermanager.getapplicationrestrictions()	Returns a Bundle containing any saved application restrictions for the context user, for the given package name
3008	Landroid/security/keychain.getcertificatechain()	Returns the X509Certificate chain for the requested alias, or null if the alias does not exist or the caller has no permission to access it
8149	Landroid/telephony/gsm/smsmessage.getoriginatingaddress()	Returns the originating address (sender) of this SMS message in String form or null if unavailable
10,418	Landroid/telecom/Connection.setdisconnected()	Sets state to disconnected
11,347	Landroid/net/Network.bindsocket()	Binds the specified Socket to this Network
21,193	Landroid/view/keyevent.getkeycharactermap()	Gets the KeyCharacterMap associated with the keyboard device

## 3 Experimental Design, Materials and Methods

The entire dataset generation pipeline, including feature extraction, data labelling, and dataset creation, is implemented by two Python-based tools, AMGenerator and AMExplorer, which we developed explicitly for this purpose [3]. We used these tools to generate the dataset.

**Fig. 1:**
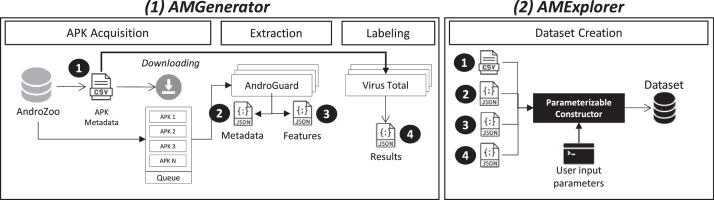
Overview of AMGenerator (1) and AMExplorer (2) tools.

Fig. 1 provides an overview of AMGenerator (1) and AMExplorer (2). AMGenerator consists of three primary modules: Acquisition, Extraction, and Labelling. Conversely, the AMExplorer tool utilizes the data and metadata generated by each AMExplorer module to construct the dataset.

The initial module accepts a list of cryptographic summaries for applications as input and proceeds to download each one. These applications were sourced from the AndroZoo repository [2]. Applications that were successfully downloaded are added to the feature extraction queue. If the download cannot be completed, the application is deleted and will not be used as a sample in the dataset.

During the second stage of the pipeline, features are extracted using AndroGuard [4], a specialized tool and Python library designed to extract information from APK files. Each Android app is unpacked during extraction, leading to an intermediate code for analysis and extraction (e.g., building a graph of API calls). This module produces a data file containing information on the application's attributes, which is then forwarded to the dataset-building data queue.

The Labelling module acquires information about an APK from VirusTotal [5], enabling users to determine later whether an APK is benign or malicious. VirusTotal provide dozens of scanners. Users can define their own threshold (e.g., 5 scanners) for labelling an APK as malware. For instance, as of July 2023, VirusTotal provides access to over 60 scanners for APK categorization. Each scanner service yields a result for the APK, and given potential disagreements amongst different scanners, a decision-making process (e.g., majority rule) becomes necessary. Users can label samples based on arbitrary thresholds or leverage solutions such as Maat [6], which provides means for automatically labelling samples based on VirusTotal's metadata.

Lastly, the AMExplorer tool (Fig. 1) explores and compiles output data from the three tool generator modules to create the final dataset. The dataset includes binary values (0 or 1) for commonly used Android malware classification features, such as permissions, intents, and API calls.

## Ethics statement

The work did not involve any human subject or animal experiments.

## CRediT authorship contribution statement

**Hendrio Bragança:** Conceptualization, Methodology, Investigation, Data curation, Formal analysis. **Vanderson Rocha:** Conceptualization, Methodology, Software, Investigation, Data curation, Writing – review & editing. **Lucas Barcellos:** Conceptualization, Methodology, Software, Investigation, Data curation. **Eduardo Souto:** Conceptualization, Supervision, Writing – review & editing. **Diego Kreutz:** Conceptualization, Resources, Software, Supervision, Writing – review & editing. **Eduardo Feitosa:** Conceptualization, Supervision, Writing – review & editing.

## Data Availability

MH-100K (Original data) (Figshare)
